# Considering age when making treatment decisions in the ICU: too little, too much, or just right?

**DOI:** 10.1186/s13054-014-0483-3

**Published:** 2014-09-09

**Authors:** William J Ehlenbach

**Affiliations:** Department of Medicine, University of Wisconsin School of Medicine and Public Health, 5245 MFCB, MC 2281, 1685 Highland Avenue, Madison, WI 53705 USA

## Abstract

There are a number of studies providing evidence that age is associated with treatment decisions for critically ill adults, although most of these studies have been unable to fully account for both prehospital health status and severity of acute illness. In the previous issue of *Critical Care*, Turnbull and colleagues present a well-executed study analyzing data from a prospective cohort study of critically ill patients with acute respiratory distress syndrome to investigate the association between age and new limitations in life-sustaining therapy. They report a strong association between age and new limitations in life support in this cohort, even after adjusting for comorbidities, prehospital functional status, and severity of illness including daily organ dysfunction scores. Their results demonstrate that decisions about the goals of care and the ongoing use of life-sustaining treatments should be viewed as dynamic and responsive to events occurring during critical illness. This study raises the important question about the contributors to this association, and the authors raise the possibility that physician or surrogate bias may be contributing to decisions for older patients. While this is unlikely to be the only contributor to the association between age and end-of-life decisions, the mere possibility should prompt reflection on the part of clinicians caring for critically ill patients.

Decisions about the use or withdrawal of life-sustaining therapies (LSTs) are made every day in ICUs around the world. Shared decision-making has been strongly endorsed by critical care societies as a framework for making these decisions. In this model, the patient (or his surrogate) and clinicians collaborate to make decisions regarding which therapies to use, withhold, or withdraw, informed by the patient’s values and preferences as well as the clinician’s scientific knowledge about indications, risks, and prognosis.

While it may be easy to differentiate a clinician appropriately considering metastatic malignancy when making a recommendation regarding LST from a clinician inappropriately factoring a patient’s poverty into that recommendation, the question of age is more challenging. In the previous issue of *Critical Care*, Turnbull and colleagues provide evidence of an association between age and decisions to limit LST in a prospective cohort study of critically ill patients with acute respiratory distress syndrome, including those with mild acute respiratory distress syndrome referred to by the authors using its former name, acute lung injury [1].

In the study, subjects were followed until one of three outcomes occurred: cardiac arrest, discharge from the ICU, or a new limitation in LST (which included the following orders: do not resuscitate, do not reintubate, no vasopressors, no hemodialysis, do not escalate care, or comfort care only) [1]. Turnbull and colleagues report that in this relatively young cohort of individuals without any limitations in life support at enrollment, every 10 years of age was associated with a 32% greater likelihood of new limitations in LST even when adjusting for comorbid disease, preadmission functional status, and initial severity of illness. The strength of this relationship was reduced by one-quarter when the authors additionally adjusted for daily organ dysfunction scores, suggesting that part of the association is driven by the trajectory of acute illness. While one can argue that a do-not-resuscitate order is different from the other limitations they studied, the fact that only 14% of those with any new limitation survived to ICU discharge and that nearly 70% of the deaths occurred after withdrawal of LST underscores the fact that these limitations were strongly associated with outcomes.

This study adds to existing literature demonstrating an association between older age and decisions in the ICU [2,3], and these data raise the possibility that age directly factored into clinicians’ or surrogates’ decisions to limit the use of LST. The study implies an important question: how much should age factor into clinician recommendations? There is mixed evidence regarding the degree to which age affects mortality from critical illness. Age is strongly associated with mortality in crude analyses, but many studies show that accounting for severity of acute illness, burden of chronic disease, and premorbid functional status attenuates or eliminates age as an independent factor [4]. Age is thus better thought of as a modifier of the association between severity of chronic and acute illness and mortality than as an independent predictor. Short-term survival is probably not the most important factor considered when making treatment decisions, and older age may increase the likelihood of less favorable post-ICU outcomes such as persistent cognitive impairment [5,6], long-lasting physical functional limitations, including the loss of independence [7,8], or psychological symptoms [9]. Clinicians should be aware that a patient’s notion about what constitutes an acceptable outcome may change with aging, and that age may affect one’s willingness to accept the risks that a therapy will result in a less desirable outcome, considered in the context of the burdens of current and future treatments [3,10]. However, clinicians should not make assumptions about the preferences of their older patients based on age alone [11].

The question of what accounts for the association between age and LST limitation remains. It is possible that age-variant preferences are the driver of these differences, or that physician or surrogate bias plays a role. These are not the only possibilities, however. Mortality in the group of acute respiratory distress syndrome patients without any LST limitation in this study was relatively low (16%). In fact, a higher proportion of patients in the limitation group suffered cardiac arrest while still on LST (28%) than died in the ‘no limitation’ group. This raises the possibility that clinicians’ recommendations regarding LST in this group, assuming that such recommendations contributed to new limitations, were influenced by accurate assessments of prognosis in a way that was not fully captured using severity of illness and daily organ dysfunction scores in regression models.

The association between age and decisions to limit LST in the ICU seen in this and other studies is worthy of further investigation. In particular, a better understanding of the association between age and treatment preferences, and of the way that patient age might inappropriately affect clinician recommendations, is needed. This evidence should also prompt ICU clinicians to be cognizant of the risk of weighing patient age too strongly when considering treatment options.

## Abbreviation

LST: Life-sustaining therapy
